# Diagnostic and Prognostic Value of Right Ventricular Fat Quantification from Computed Tomography in Arrhythmogenic Right Ventricular Cardiomyopathy

**DOI:** 10.3390/jcm13133674

**Published:** 2024-06-24

**Authors:** Valentina Faga, María Ruiz Cueto, David Viladés Medel, Zoraida Moreno-Weidmann, Paolo D. Dallaglio, Carles Diez Lopez, Gerard Roura, Jose M. Guerra, Rubén Leta Petracca, Joan Antoni Gomez-Hospital, Josep Comin Colet, Ignasi Anguera, Andrea Di Marco

**Affiliations:** 1 Cardiology Department, Bellvitge University Hospital, Hospitalet de Llobregat, 08907 Barcelona, Spain; 2 BIOHEART Group, Cardiovascular, Respiratory and Systemic Disease and Cellular Aginf Program, Institut d’Investigación Biomèdica de Bellvitge (IDIBELL), Hospitalet de Llobregat, 08907 Barcelona, Spain; 3 Cardiology Department, Hospital de la Santa Creu i Sant Pau, 08041 Barcelona, Spain; 4 Cardiac Imaging Unit, Hospital de la Creu Blanca, 08034 Barcelona, Spain; 5 Centro de Investigación Biomédica en Red en Enfermedades Cardiovasculares (CIBERCV), 28029 Madrid, Spain; 6 Institut de Recerca Sant Pau, 08041 Barcelona, Spain; 7 Faculty of Medicine, Universitat Autònoma de Barcelona, 08193 Barcelona, Spain; 8 Innovation, Research and Universities Department, Gerència Territorial Metropolitana Sud, Institut Català de la Salut, Hospitalet de Llobregat, 08907 Barcelona, Spain; 9 Department of Clinical Sciences, School of Medicine, University of Barcelona, 08007 Barcelona, Spain

**Keywords:** arrhythmogenic right ventricular cardiomyopathy, cardiac computed tomography, ventricular arrhythmias, sudden death, myocardial fibrofatty replacement

## Abstract

**Background:** In arrhythmogenic right ventricular cardiomyopathy (ARVC) non-invasive scar evaluation is not included among the diagnostic criteria or the predictors of ventricular arrhythmias (VA) and sudden death (SD). Computed tomography (CT) has excellent spatial resolution and allows a clear distinction between myocardium and fat; thus, it has great potential for the evaluation of myocardial scar in ARVC. **Objective:** The objective of this study is to evaluate the feasibility, and the diagnostic and prognostic value of semi-automated quantification of right ventricular (RV) fat replacement from CT images. **Methods:** An observational case–control study was carried out including 23 patients with a definite (19) or borderline (4) ARVC diagnosis and 23 age- and sex-matched controls without structural heart disease. All patients underwent contrast-enhanced cardiac CT. RV images were semi-automatically reconstructed with the ADAS-3D software (ADAS3D Medical, Barcelona, Spain). A fibrofatty scar was defined as values of Hounsfield Units (HU) <−10. Within the scar, a border zone (between −10 HU and −50 HU) and dense scar (<−50 HU) were distinguished. **Results:** All ARVC patients had an RV scar and all scar-related measurements were significantly higher in ARVC cases than in controls (*p* < 0.001). The total scar area and dense scar area showed no overlapping values between cases and controls, achieving perfect diagnostic performance (sensitivity and specificity of 100%). Among ARVC patients, 16 (70%) had experienced sustained VA or aborted SD. Among all clinical, ECG and imaging parameters, the dense scar area was the only one with a statistically significant association with VA and SD (*p* = 0.003). **Conclusions:** In ARVC, RV myocardial fat quantification from CT is feasible and may have considerable diagnostic and prognostic value.

## 1. Introduction

Arrhythmogenic right ventricular cardiomyopathy (ARVC) is a relatively rare disease with an estimated prevalence of 1:2000 to 1:5000 [1]. Fibrofatty replacement of the right ventricular (RV) myocardium is the major hallmark of ARVC; however, a significant proportion of patients can also display abnormalities in the left ventricle (LV) [2]. The genetic basis of ARVC is usually related to desmosomal genes such as *PKP2, DSP, DSG2, DSC2* and *JUP*, although pathogenic or likely pathogenic variants in other genes such as *TMEM43*, *DES* and *PLN* can also cause the disease [3].

Patients with ARVC are at high risk for ventricular arrhythmias (VA) and sudden death (SD); actually, despite being a relatively rare disease, ARVC is an important cause of sudden death among young adults and athletes [4]. Therefore, risk stratification for VA and SD is a cornerstone in the management of this disease.

In other cardiomyopathies, such as non-ischemic dilated cardiomyopathy, non-invasive scar characterization of the LV with cardiac magnetic resonance (CMR) and late gadolinium enhancement (LGE) has an important role in differential diagnosis and in risk stratification [5].

By contrast, non-invasive scar analysis in the RV is not included either among the 2010 Task Force diagnostic criteria of ARVC [6] or in the ARVC risk score calculator employed to predict VA and SD [7], likely because CMR has shown limited accuracy to detect RV scars [8,9,10].

Contrast-enhanced computed tomography (CT) has excellent spatial resolution and allows a clear distinction between myocardium and adipose tissue. One prior report has suggested a potential diagnostic usefulness of RV myocardial fat quantification from CT images in ARVC [11].

We performed semi-automatic quantification of RV fat from CT images with the aim of confirming its feasibility and assessing its diagnostic value and its potential association with VA in ARVC patients.

## 2. Methods

A prospective case–control study was performed at three hospitals in Barcelona, Spain. The study was approved by the ethical committee at Bellvitge University Hospital and all patients signed the informed consent form. The study is in line with the Declaration of Helsinki. Data will be available upon reasonable request.

Cases were consecutive patients with a definite or borderline ARVC diagnosis, according to the 2010 criteria [6], under follow-up at our hospitals. Controls were patients without structural heart disease who underwent cardiac CT prior to atrial fibrillation ablation. Controls were matched for age (+/− 5 years) and sex and were selected if the CT showed adequate enhancement of the blood pool in the RV.

Genetic testing in cases was performed using NGS panels of >50 genes, including all genes which have been related to ARVC. All genetic variants were interpreted according to the standards and guidelines set forth by the American College of Medical Genetics and Genomics and the Association for Molecular Pathology [12].

The following events were considered as episodes of sustained VA or aborted SD: appropriate implantable cardioverter defibrillator (ICD) therapies, sustained monomorphic ventricular tachycardia (SMVT) and resuscitated cardiac arrest.

### 2.1. Cardiac CT

Cardiac multidetector CTs were carried out prospectively between 2019 and 2023. These exams were performed using a contrast-enhanced, prospective ECG-triggered method with a wide-coverage scanner (320-row scan Aquilion One; Canon Medical Systems, Otawara, Japan). The study protocol was initiated with a topogram to determine the limits of cardiac volume acquisition, which were generally set between the tracheal carina, the trachea and the diaphragmatic domes. The radiation parameters were adjusted according to the morphological characteristics of each patient: tube voltage and current settings ranged from 100 to 120 kV and 250 to 600 mA, respectively. Beta-blockers were administered unless contraindicated to every patient with a heart rate higher than 60 beats per minute. Images were acquired during the first pass of iodinated contrast media (iobitridol − Xenetix 350 mg/mL, Guerbet, Aulnay-sous-Bois, France). To achieve optimal enhancement of the RV chamber, a biphasic bolus method was applied; specifically, 1 mL/kg of contrast media followed by 80 mL of a 50:50 mixture of iodinated contrast material and saline, both delivered at a rate of 5 mL/sec [11].

The study utilized a bolus-track technique with a region of interest located in the descending aorta, and images were reconstructed at end-diastole in a stack of contiguous 0.5 mm thick short-axis sections encompassing the entire RV (typical in-plane pixel size, 0.5 × 0.5 mm), as well as functional reconstructions every 10% of the R-R interval with a thickness of 1.5 mm without overlap.

Typical acquisition parameters included a gantry rotation time of 275 msec (temporal resolution of 135 msec), collimation of 320 × 0.5 mm, tube voltage between 100 and 120 kV, and a typical tube current of 600 mAs with a dose modulation protocol using Adaptive Iterative Dose Reduction 3D (AIDR3D). The retrospectively acquired scans were reconstructed with a slice thickness/interval of 0.5/0.25 mm using an FC03 algorithm and AIDR3D.

Cine CT scans, analysing RV and LV volumes and function, were performed in 11 ARVC patients who either had never had a CMR or had it performed >3 years before the CT scan. In these 11 ARVC patients, RV and LV volumes and function were derived from CT analysis, while in the rest of the patients, these parameters were obtained from CMR. This approach was considered appropriate in view of the high correlation between CMR and CT when evaluating ventricular volumes and function [13].

The mean effective radiation dose was 9.6 mSv (SD 4) for standard CT scans and 15 mSv (SD 4) for cine CT. All images were analysed by a level 3 cardiac CT reader.

### 2.2. Quantification of RV Fat

The ADAS-3D software (https://www.adas3d.com/, ADAS3D Medical, Barcelona, Spain) was used to build a 3D model of the RV, incorporating a semi-automatic quantification of myocardial fat. Of note, in the present manuscript, fat/fibrofatty infiltration and scar will be used as interchangeable terms.

**Figure 1:**
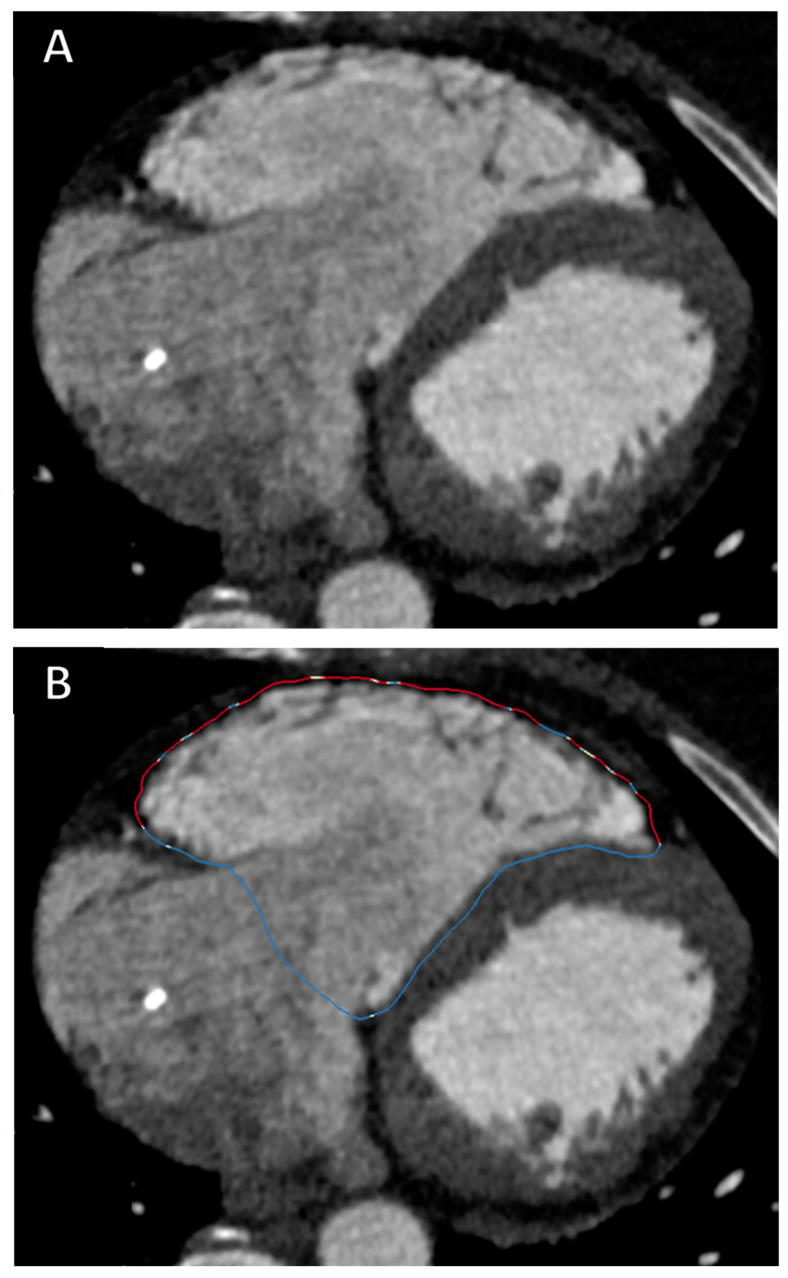
Panel (**A**) shows CT image of a patient with ARVC; the RV is dilated plus wall thinning and hypoattenuations of the RV lateral wall are clearly visible. Panel (**B**) shows the RV border drawn by ADAS-3D used to calculate scar parameters. The colours of the border indicate the type of myocardial tissue: blue is healthy myocardium, red is dense scar, white-orange is border zone.

After identifying specific hallmarks in the RV and in the left ventricle (the LV apex, the aortic valve, the mitral valve and the tricuspid valve), the ADAS-3D software elaborates a 3D reconstruction of heart chambers based on the signal intensity of the blood pool (which can be adjusted by the operator). Once this volume rendering is completed, the endocardial border of the right ventricle is automatically drawn and, if needed, it can be manually adjusted. The RV endocardial border can be verified and adjusted using any plane, and the same point can be simultaneously visualized in three orthogonal planes in order to optimize the exact correspondence between the ADAS-3D border and the true endocardial border. In this study, after obtaining the automatic endocardial border of the RV, we adjusted it to make sure it perfectly followed the frontier between the RV myocardium and the blood pool. Once the adjustment of the RV endocardial border was finalized, the software automatically dilated such a border 1 mm towards the epicardium, to be sure to avoid the blood pool while remaining within the myocardium. This “dilated” border was used to generate the 3D surface of the RV. All the measurements provided in this manuscript were obtained from the 3D surface of the RV based on the RV border automatically drawn by the software 1 mm outside of the endocardial RV border (see Figure 1).

One operator, blinded to the clinical data of the patients, performed all ADAS reconstructions. A second operator processed 10 patients (5 cases and 5 controls) to assess interobserver variability.

ADAS-3D records the Hounsfield Units (HU) of every pixel in the RV 3D surface. Based on the values of the HU, the software can classify three types of tissue: the healthy myocardium, border zone (otherwise called heterogeneous scar) and dense scar. These types of tissue are visually represented with different colours in the 3D surface of the RV: blue for healthy myocardium, a range of colours between white and orange for border zone and red for dense scar.

**Figure 2:**
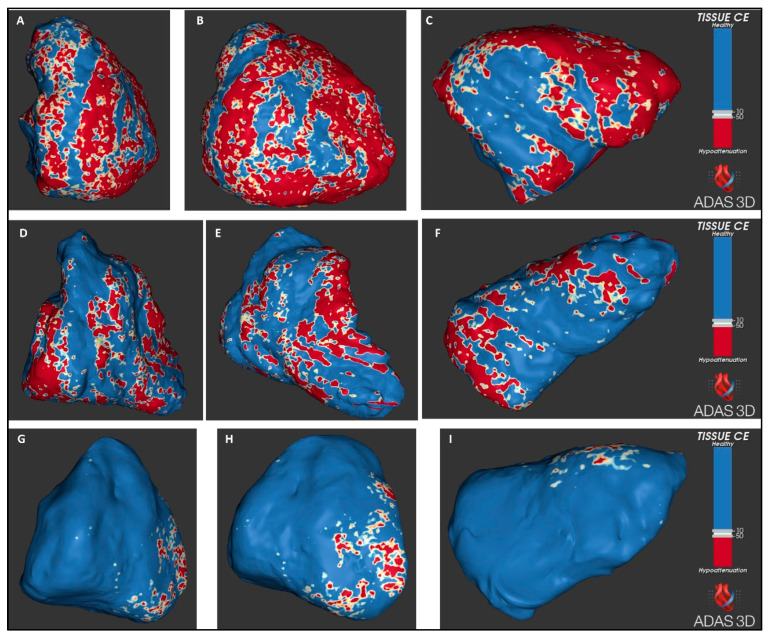
Examples of RV reconstructions with ADAS 3D in two patients with definite diagnosis of ARVC, one with (panels (**A**–**C**)) and the other without (panels (**D**–**F**)) prior to VA. Panels (**G**–**I**) are from a control. Right anterior oblique views (**A**,**D**,**G**), antero-posterior views (**B**,**E**,**H**) and inferior views (**C**,**F**,**I**).

The range of HU for fat is generally considered between −50 and −150 [14], while the normal myocardium generally displays +50 to +100 HU [15,16]. In line with one prior report, we considered scar for values <−10 HU [16]. Additionally, we used −50 HU as the cut-off for dense scar, considering that values <−50 HU would correspond to a clear predominance of fat infiltration. Thus, values between −10 HU and −50 HU identified the border zone and values <−50 HU identified the dense scar. ADAS-3D reconstructions of the RV border are shown in Figure 2.

In order to analyse RV scar distribution, the RV was divided into 8 segments, similarly to one prior report [11]: three basal segments (anterior, lateral and inferior), three mid segments (anterior, lateral and inferior), apex and septum. The basal anterior segment included the right ventricular outflow tract (RVOT). Lateral segments included the acute angle.

### 2.3. Statistical Analysis

All statistical analyses were performed by using STATA RELEASE 12 software (StataCorp LP, College Station, TX, USA). Continuous variables are presented as means and SD or medians and interquartile range, depending on the normality of their distribution as assessed with the Shapiro–Wilk test. Comparisons between groups were undertaken using the Student’s *t*-test or the Wilcoxon rank sum test for continuous variables and the chi-square test or the Fisher exact test for categorical variables. The agreement between two operators was evaluated with the interclass correlation coefficients (ICCs).

For continuous variables, the best cut-off to diagnose ARVC was identified with the Liu method, maximising the product of specificity and sensitivity [17]. The performance of ADAS-3D parameters for the diagnosis of ARVC was evaluated calculating the sensitivity, specificity and efficiency of the best cut-off value for each parameter. The 95% CIs of the sensitivity and specificity were calculated with the Wilson’s method. Efficiency is defined as the percentage of correct classifications by a diagnostic test and it is calculated as follows: 100 × (true negatives + true positives)/all cases. Finally, the area under the ROC curve (AUC) was also calculated to evaluate the global diagnostic performance.

The correlation between continuous variables was evaluated using the Pearson or, when appropriate, the Spearman correlation test.

Differences were considered statistically significant at the two-sided *p* < 0.05 level.

## 3. Results

### 3.1. Baseline Characteristics

A total of 46 patients were included, 23 cases and 23 controls. Interobserver agreement was high: the ICC was 0.98 for the total scar area, 0.97 for the border zone area and 0.96 for the dense scar area. The baseline characteristics of ARVC cases are detailed in Table 1. The mean age was 53 and most of the patients (65%) were males. A pathogenic or likely pathogenic genetic variant was found in 10 patients, in most cases (60%) in the *PKP2* gene. The mean right ventricular ejection fraction (RVEF) was 37%, while only two patients had a left ventricular ejection fraction (LVFE) <50%. Among the ARVC cases, 19 (83%) had a definite diagnosis and 4 a borderline diagnosis. Those with a borderline diagnosis had a trend towards smaller RV volumes and better RV function; they also had a significantly lower number of precordial leads with inverted T waves (1.3 vs. 3.7, *p* = 0.01).

### 3.2. Comparison between ARVC Patients and Controls

Cases and controls had similar ages (*p* = 0.77). As shown in Table 2, ARVC patients had a significantly larger RV indexed area (153 cm^2^/m^2^ vs. 96 cm^2^/m^2^, *p* < 0.001); however, there was a certain degree of overlap between the values of the RV indexed area observed in cases and those of controls (minimum indexed area in cases was 118 cm^2^/m^2^ and maximum RV indexed area in controls was 131 cm^2^/m^2^).

**Figure 3:**
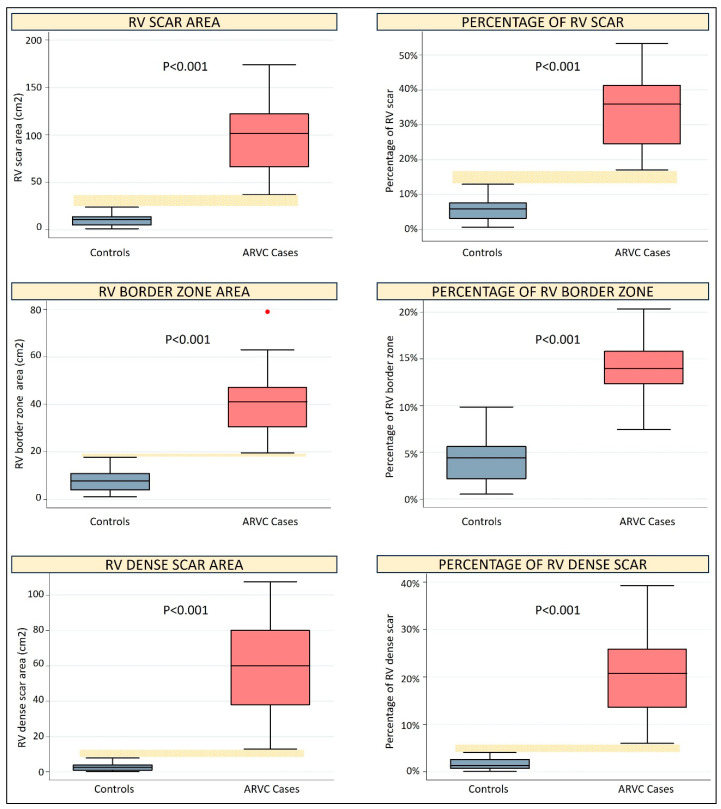
Box plot graphs comparing scar parameters between controls and ARVC cases. The yellow dotted horizontal bar highlights the difference between the minimum value of ARVC cases and the maximum value of controls, whenever this difference is greater than zero.

All RV scar parameters were significantly higher in ARVC cases as compared to controls (*p* < 0.001 for all comparisons, see Table 2, Figure 2 and Figure 3). Of note, the values of the non-indexed, indexed and percentage total scar area and dense scar area, as well as the non-indexed border zone area, exhibited no overlapping between cases and controls, indicating perfect diagnostic discrimination. As shown in Table 3, the best cut-offs of all these parameters had 100% sensitivity, 100% specificity and an AUC of 1 for the diagnosis of ARVC.

**Figure 4:**
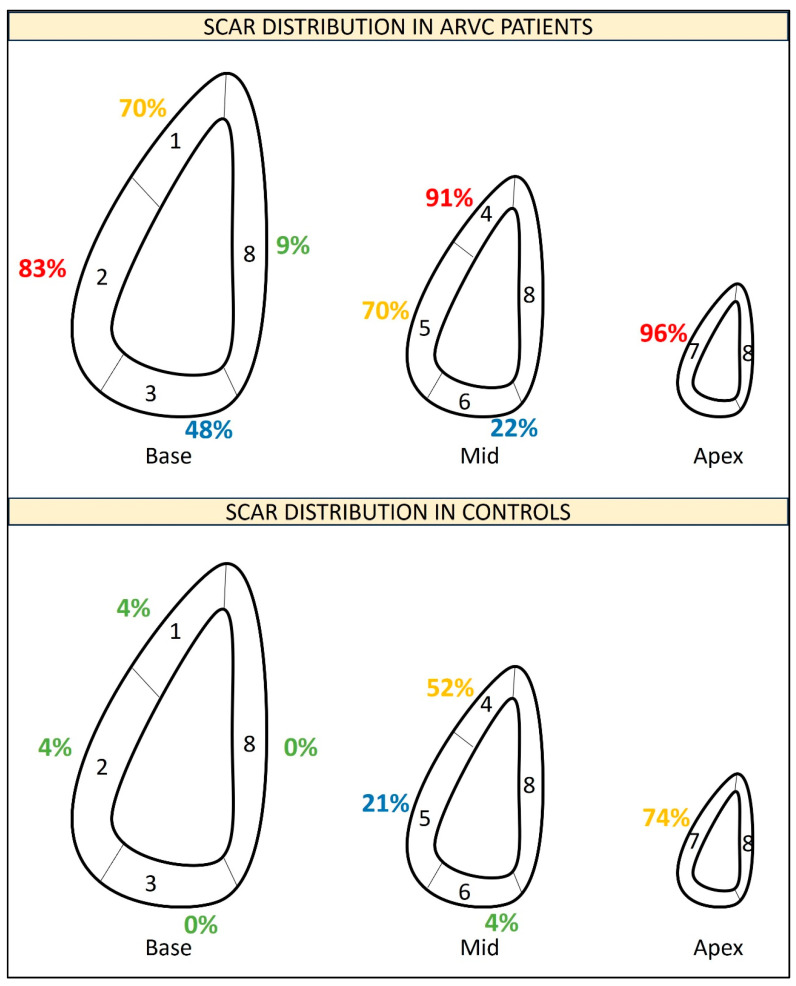
Segmentation of the RV into 8 segments. The percentages of patients with a scar in every RV segment are detailed.

As shown in Supplementary Table S1 and in Figure 4, in ARVC patients, the scar was mainly located in the RV apex (96% of patients) followed by the mid anterior RV wall (91% of cases), the basal lateral RV wall (83% of patients), the basal anterior RV wall and the mid lateral RV wall (both present in 70% of patients). However, the RV apex and the mid anterior RV wall were also the most frequent locations of fat infiltration in controls (present in 74% and 52% of controls, respectively). As shown in Supplementary Table S2, scars in the RV apex and in the mid anterior RV wall had low specificity (26% and 48%, respectively) to diagnose ARVC. Among the parameters of scar location, scars in the inferior basal RV segment achieved 100% specificity for ARVC diagnosis and scars in any of the basal segments achieved the highest overall diagnostic performance for ARVC (sensitivity, specificity and efficiency of 91%).

When patients with a borderline diagnosis and their correspondent controls were excluded, all scar parameters exhibited no overlapping between cases and controls (Supplementary Table S3).

A separate analysis including only patients with a borderline ARVC diagnosis and their correspondent controls showed that, despite the low number of patients, there were statistically significant differences between cases and controls in all scar parameters and there was no overlapping in any scar value between cases and controls (Supplementary Table S4).

### 3.3. Correlates of RV Fat in ARVC Patients

These results are detailed in Supplementary Table S5. There were no significant differences in indexed RV scar areas between males and females (*p* = 0.98 for total scar, *p* = 0.58 for border zone and *p* = 0.83 for dense scar); no correlation was also found between age and any of the RV scar parameters.

Statistically significant negative linear correlations of moderate strength (r values between −0.4 and −0.58) were found between scar areas/scar percentages and RVEF. By contrast, most scar areas/scar percentages had a non-significant correlation with TAPSE. Statistically significant positive linear correlations of moderate or high strength (r between 0.48 and 0.92) were observed between scar areas and RV end-diastolic volume (RVEDV); by contrast, among RV scar percentages, only the percentage of the RV border zone significantly correlated with RVEDV.

Among ECG variables, there was a statistically significant positive correlation of moderate strength (r values between 0.43 and 0.69) between the number of negative T waves in precordial leads and all scar areas, the percentage of RV scar and the percentage of RV dense scar. By contrast, there was no significant difference in any of the RV scar parameters between patients with or without epsilon wave.

### 3.4. Correlates of RV Fat in Controls

There was a statistically significant correlation of moderate strength (r 0.47, *p* = 0.02) between age and percentage of total fat infiltration in controls. By contrast, no significant correlation was found between body mass index and total fat infiltration (*p* = 0.09).

### 3.5. Ventricular Arrhythmias

Among ARVC cases, 16 (70%) had experienced sustained ventricular arrhythmias: the first arrhythmic episode had been resuscitated cardiac arrest for two patients, sustained monomorphic ventricular tachycardia for 12 cases and appropriate ICD therapies in the remaining two patients.

The characteristics of patients with or without VA are detailed in Table 4. There was a trend towards a higher prevalence of VA in males than in females (80% vs. 50%, *p* = 0.18) and in patients with a definite as compared to those with a borderline diagnosis (79% vs. 25%, *p* = 0.07). There were no significant differences between patients with or without VA in terms of ECG parameters, RVEF and RVEDV.

Among scar-related parameters, the RV border zone had no association with VA, either when considered as a border zone area (*p* = 0.76) or when considered as a percentage of the border zone (*p* = 0.83). The total RV scar area showed a trend towards an association with VA: the mean RV scar area was 111 cm^2^ in patients with VA and 83 cm^2^ in those without VA (*p* = 0.09), and the percentage of the RV scar was 37% in those with VA and 28% in those without VA (*p* = 0.05). RV dense scar was the only parameter achieving a significant association with VA: the RV dense scar area was 69 cm^2^ in patients with VA and 43 cm^2^ in those without VA (*p* = 0.03), and the percentage of dense scar in the RV was 23% in those with VA vs. 15% in those without VA (*p* = 0.03).

## 4. Discussion

The present work shows the feasibility of quantifying RV myocardial fat using CT images processed with ADAS-3D. Such quantification of RV fat allows a clear differentiation between ARVC patients and controls. In addition, the amount of myocardial RV fat infiltration, especially dense scar below −50 HU, is associated with the occurrence of sustained VA among ARVC patients.

### 4.1. Fat Quantification for ARVC Diagnosis

The diagnosis of ARVC can be challenging. Fibrofatty myocardial replacement in the RV is the main histological feature of ARVC [18]; however, non-invasive evaluation of such substrate is difficult. In addition, fibrofatty infiltration in the RV has been described in healthy individuals [19].

CMR is the gold standard for myocardial tissue characterization of the LV, but it faces important limitations in the RV, mainly due to sub-optimal spatial resolution. The prevalence of RV fat under CMR in ARVC patients has been highly variable across studies, ranging from 22% to 100% [20]. In addition, qualitative evaluation of RV fat by CMR has been shown to be a major cause of ARVC misdiagnosis [10] and, by consequence, it is considered non-reliable.

LGE evaluation in the thinned RV myocardium may also be challenging: several studies have documented a low prevalence of RV LGE in ARVC, between 15% [9] and 30% [8]. By contrast, a small report including eight ARVC patients claimed that 88% had LGE in the RV [21]. In the present study, the prevalence of RV LGE among ARVC patients was 38%, confirming the low sensitivity of this parameter in ARVC. The lack of reproducibility and the limited sensitivity has prevented the inclusion of RV LGE in 2010 Task Force diagnostic criteria.

The quantification of RV myocardial fat with a CT scan may help to fill this gap. The quantitative evaluation of RV myocardial hypoattenuations obtained from CT scans analysed with ADAS-3D was found to correlate with epicardial voltage and epicardial scars in ARVC [22], confirming the ability of RV fat quantification to precisely assess the substrate in ARVC.

The potential diagnostic usefulness of RV fat quantification from contrast-enhanced cardiac CT was initially demonstrated by Cochet and colleagues, using another type of software for CT reconstruction [11]. Comparing 36 ARVC patients with 36 controls without structural heart disease and 36 controls with ischemic cardiomyopathy, they found that RV fat infiltration (defined as <−10 HU) had superior diagnostic performance as compared to RVEDV, achieving an AUC of 0.96 [11]. In the present work, we have confirmed and further expanded these prior data. In our population, both total fat infiltration (defined as <−10 HU) and dense scar (<−50 HU) showed an excellent diagnostic performance for ARVC, with 100% sensitivity and specificity. The border zone area (also called heterogeneous tissue, with values between −10 and −50 HU) also had a very good diagnostic ability, although slightly lower as compared to the other two scar-related parameters. In light of these data, and considering also that scar <−50 HU was minimal (1.6% of RV area) and had low variability (SD 1%) in controls, scar <−50 HU might be the best target for diagnostic purposes.

Of note, when the analysis was restricted to the four patients with a borderline ARVC diagnosis and their correspondent controls, all scar parameters showed significant differences between cases and controls (despite the low number of patients) and there was no overlapping in the values of any scar parameter between cases and controls; thus, RV fat quantification might be a useful tool to clarify the diagnosis in patients who are classified as borderline ARVC according to the 2010 ARVC criteria.

Finally, scar variables had significant linear correlation with other parameters of RV size and function, as well as with the number of negative T waves in precordial leads, yet this correlation was of moderate strength in most cases; thus, RV fat quantification may be complementary to other evaluations in ARVC patients, providing a specific and differential information.

These findings may have important clinical and research implications. Future studies should evaluate the reproducibility of fat quantification and might explore the optimal cut-off to define fat infiltration, possibly through validation against invasive electroanatomical mapping. If our results are confirmed in larger studies, fat quantification from CT scans might be included among the diagnostic criteria for ARVC.

### 4.2. Fat Distribution in the RV

The distribution of fibrofatty infiltration in ARVC follows certain patterns: it spreads from the epicardium to the endocardium and it usually involves specific areas of the RV free wall while sparing the septum [18]. A recent study using spatial transcriptomics has provided new molecular insights on these features of the disease, observing enhanced Zinc finger and BTB domain-containing protein 11 (*ZBTB11*) expression in areas of active remodelling and new fibrofatty infiltration [23]. Therefore, specific molecular pathways may be involved not only in the occurrence of fibrofatty infiltration but also in its specific localization.

The classical concept of the “triangle of dysplasia” suggested that the most frequent areas of fat infiltration in ARVC are the basal inferior wall, the basal anterior wall and the apex [18]. Our results are partially in line with these classical observations. In our cohort, almost all ARVC patients had fibrofatty infiltration in the RV apex; however this finding lacked specificity since the RV apex was also the most frequent location of hypoattenuations in controls. We observed that fat infiltration in the basal RV segments achieved the highest specificity for ARVC diagnosis; however, the most frequent location in the basal RV was the basal lateral RV wall rather than the inferior wall (the latter was anyway present in 48% of ARVC patients). Of note, a similar finding was also reported by Cochet and colleagues [11]. It should be underscored that, in our segmentation of the RV, the lateral wall included the acute angle and this fact may have contributed to the high prevalence of fat infiltration in the basal lateral RV segment. We also observed a high prevalence of fat infiltration in the mid anterior segment, again in line with the prior data about fat quantification in ARVC [11].

Among controls, the mid anterior RV wall and RV apex were the most frequent locations of fat infiltration in the RV, similarly to what was observed by Cochet and colleagues. Although the RV border used in the present work was expanded just 1 mm from the RV endocardial border, we cannot exclude that part of these abnormalities might be due to the detection of epicardial fat, since the anterior and apical segments of the RV are among the thinnest ones [24]. In patients without structural heart disease, progressive fat infiltration of the RV may also be part of a natural aging process; in this respect, our observation that fat infiltration in controls correlates with age is in line with prior histological observations [19].

### 4.3. Fat Quantification and Ventricular Arrhythmias

ARVC is associated with a considerable risk of VA and SD. Indeed, risk stratification for VA and SD is the cornerstone of ARVC management and is crucial for adequately allocating primary prevention ICDs. Recently, a risk calculator was developed and validated [7,25]. RVEF is the only imaging parameter included in this calculator. While it is intuitive that RVEF reflects, at least in part, the extent of RV scar, our data show that the correlation between RVEF and scar, although statistically significant, is of moderate strength only. In the case of non-ischemic dilated cardiomyopathy, it is well recognized that LVEF alone has limited sensitivity and specificity to predict VA and SD [26]; by contrast, direct LV scar assessment with LGE analysis has consistently demonstrated a strong and independent association with VA and SD [27].

Since myocardial scar is the main substrate for VA in most cardiomyopathies, including ARVC, it is likely that direct scar assessment and quantification may hold the highest predictive ability for VA and SD. Unfortunately, non-invasive scar evaluation in ARVC has thus far been limited by the low sensitivity and reproducibility of CMR in these patients. Indeed, in our population, dense scar (<−50 HU) was the only parameter (among all the clinical, ECG and imaging variables evaluated) with a statistically significant association with VA and aborted SD. These are novel and extremely promising results. If confirmed in future studies, these data may contribute to a further refinement of the ARVC risk score incorporating the quantification of RV fat infiltration.

## 5. Limitations

This is an observational study; therefore, association does not equal causality. Due to the low number of cases, we could not evaluate correlations between RV scar and the genetic background. The limited sample size could account for the lack of association between well-known predictors of VA and the arrhythmic endpoint. The comparison between cases with borderline diagnosis and their correspondent controls, although providing statistically significant results consistently for all scar parameters, should be seen as exploratory and hypothesis generating given the low number of patients.

## 6. Conclusions

The quantification of RV myocardial fat from CT images is feasible and provides unique information with considerable diagnostic and prognostic value.

## Figures and Tables

**Table 1:** Baseline characteristics, comparing patients with definite and borderline ARVC diagnosis.

	All Patients	Definite Diagnosis (N = 19)	Borderline Diagnosis (N = 4)	*p*
Age (years)	53 (SD 13)	53 (SD 12)	56 (SD 17)	0.65
Sex (females)	8 (35%)	7 (37%)	1 (25%)	1
Time from diagnosis to CT (years)	4 (1–8)	4.5 (2–10)	2 (0.3–5)	0.29
Pathogenic or likely pathogenic variants	10 (43%)	9 (47%)	1 (33%)	1
Gene * PKP2 *DSG2*	6 (60%) 4 (40%)	5 (56%) 4 (44%)	1 (100%) 0 (0%)	
ICD	21 (91%)	18 (95%)	3 (75%)	0.32
Primary prevention ICD	7 (33%)	5 (28%)	2 (67%)	0.25
Any sustained VA or aborted SD	16 (70%)	15 (79%)	1 (25%)	0.07
ECG parameters				
QRS duration (ms)	109 (SD 17)	107 (SD 10)	121 (SD 34)	0.13
Number of negative T waves precordial leads	3.3 (SD 1.8)	3.7 (SD 1.7)	1.3 (SD 0.5)	0.01
Epsilon wave	11 (48%)	10 (53%)	1 (25%)	0.59
Terminal QRS activation(ms)	57 (SD 16)	57 (SD 13)	55 (SD 30)	0.8
Imaging				
TAPSE (mm)	18 (SD 5)	17 (SD 5)	21 (SD 2)	0.17
LVEF (%)	56 (SD 9)	56 (SD 9)	54 (SD 9)	0.62
iRVEDV (mL/m^2^)	132 (SD 35)	137 (SD 36)	108 (SD 22)	0.13
iRVESV (mL/m^2^)	85 (SD 33)	90 (SD 34)	63 (SD 20)	0.09
RVEF (%)	37 (SD 10)	35 (SD 10)	43 (SD 9)	0.21
RV LGE ^ƚ^	6 (38%)	5 (38%)	1 (33%)	1
ADAS scar parameters				
RV scar area (cm^2^)	102 (SD 37)	108 (SD 35)	77 (SD 37)	0.13
RV BZ area (cm^2^)	42 (SD 14)	44 (SD14)	33 (SD 14)	0.2
RV dense scar area (cm^2^)	61 (SD28)	64 (SD 28)	44 (SD 26)	0.19
Percentage of RV scar	34% (SD 10)	36% (SD9)	27% (SD 10)	0.11
Percentage of RV BZ	14% (SD 3)	14% (SD 2)	12% (SD 4)	0.13
Percentage of RV dense scar	20% (SD 9)	22% (SD 9)	15% (SD8)	0.2

CT, computed tomography; ICD, implantable cardioverter defibrillator; TAPSE, Tricuspid Annular Plane Systolic Excursion; LVEF, left ventricular ejection fraction; iRVEDV, indexed right ventricular end-diastolic volume; iRVESV, indexed right ventricular end systolic volume; RVEF, right ventricular ejection fraction; RV, right ventricular; LGE, late gadolinium enhancement; BZ, border zone. * Gene hosting the pathogenic or likely pathogenic variant. ^ƚ^ Information available for 16 patients who had CMR with LGE analysis.

**Table 2:** Scar parameters obtained with ADAS-3D from CT are compared between ARVC patients and controls.

	ARVC Patients	Controls	*p*
Age	53 (SD 13)	54 (SD 10)	0.77
Sex (females)	8 (35%)	8 (35%)	1
RV area (cm^2^)	278 (257–314)	178 (152–215)	<0.001
Indexed RV area (cm^2^/m^2^)	153 (135–171)	96 (83–108)	<0.001
RV scar area (cm^2^)	102 (SD 37)	11 (SD 6)	<0.001
Indexed RV scar area (cm^2^/m^2^)	54 (SD 18)	6 (SD 3)	<0.001
Percentage of RV scar (%)	34% (SD 10)	5.7% (SD 3)	<0.001
RV BZ area (cm^2^)	42 (SD14)	8 (SD 5)	<0.001
Indexed RV BZ area (cm^2^/m^2^)	22 (SD 7)	4 (SD 2)	<0.001
Percentage of RV BZ (%)	14% (SD 3)	4% (SD 2)	<0.001
RV dense scar area (cm^2^)	61 (SD 28)	3 (SD 2)	<0.001
Indexed RV dense scar area (cm^2^/m^2^)	32 (SD 14)	2 (SD 1)	<0.001
Percentage of RV dense scar (%)	20% (SD 9)	1.6% (SD 1)	<0.001
RV area Min–Max (cm^2^)	191–547	127–262	
Indexed RV area Min–Max (cm^2/^m^2^)	118–254	71–131	
RV scar area Min–Max (cm^2^)	37–174	1.4–24	
Indexed RV scar area Min–Max (cm^2^/m^2^)	23–88	0.7–13	
Percentage of RV scar Min–Max	17–53%	0.6–13%	
RV BZ area Min–Max (cm^2^)	20–79	1.2–18	
Indexed BZ area Min–Max (cm^2^/m^2^)	9–38	0.6–10	
Percentage of RV BZ Min–Max	7–20%	0.5–10%	
RV dense scar area Min–Max (cm^2^)	13–108	0.2–8	
Indexed RV dense scar Min–Max (cm^2^/m^2^)	8–61	0.1–4	
Percentage of RV dense scar Min–Max	6–39%	0.09–4%	

RV, right ventricular; BZ, border zone; Min–Max, minimum and maximum value.

**Table 3:** Diagnostic performance for ARVC of RV parameters obtained from ADAS-3D. Sensitivity, specificity and efficiency are calculated using the best cut-off for each parameter.

	Cut-Off	Sensitivity % (95% CI)	Specificity % (95% CI)	Efficiency %	AUC (95% CI)
RV area (cm^2^)	240.3	87 (78–96)	91 (73–98)	89	0.96 (0.91–1)
Indexed RV area (cm^2^/m^2^)	116.6	100 (86–100)	91 (73–98)	96	0.987 (0.96–1)
RV scar area (cm^2^)	30.5	100 (85.7–100)	100 (85.7–100)	100	1 (1–1)
Indexed RV scar area (cm^2^/m^2^)	18	100 (85.7–100)	100 (85.7–100)	100	1 (1–1)
Percentage of RV scar (%)	15%	100 (85.7–100)	100 (85.7–100)	100	1 (1–1)
RV BZ area (cm^2^)	18.7	100 (85.7–100)	100 (85.7–100)	100	1 (1–1)
Indexed RV BZ area (cm^2^/m^2^)	12.2	96 (79–99)	100 (85.7–100)	98	0.998 (0.99–1)
Percentage of RV BZ (%)	10.4%	96 (79–99)	100 (85.7–100)	98	0.996 (0.99–1)
RV dense scar area (cm^2^)	10.5	100 (85.7–100)	100 (85.7–100)	100	1 (1–1)
Indexed RV dense scar area (cm^2^/m^2^)	6.2	100 (85.7–100)	100 (85.7–100)	100	1 (1–1)
Percentage of RV dense scar (%)	5%	100 (85.7–100)	100 (85.7–100)	100	1 (1–1)

RV, right ventricular; BZ, border zone.

**Table 4:** Comparison of demographic, clinical, ECG, imaging and ADAS variables between patients with and without ventricular arrhythmias.

	No VA (N = 7)	VA (N = 16)	*p*
Age at diagnosis	50 (SD 15)	47 (SD 14)	0.66
Sex (female)	4 (57%)	4 (25%)	0.18
Definite diagnosis	4 (57%)	15 (94%)	0.07
Pathogenic or likely pathogenic variant	4 (57%)	7 (44%)	0.67
Epsilon wave	4 (57%)	7 (44%)	0.67
Number of negative T waves in precordial leads	2.7 (SD 1.5)	3.5 (SD 2)	0.36
LVEF (%)	62 (SD 7)	59 (SD 10)	0.44
RVEF (%)	36 (SD 12)	37 (SD 10)	0.82
iRVEDV (mL/m^2^)	126 (SD 46)	136 (SD 30)	0.57
iRVESV (mL/m^2^)	83 (SD 46)	87 (SD 28)	0.77
RV scar area (cm^2^)	83 (SD 45)	111 (SD 30)	0.09
Indexed RV scar area (cm^2^/m^2^)	44 (SD 20)	59 (SD 15)	0.06
Percent of RV scar	28% (SD 9)	37% (SD 9)	0.05
Border zone area (cm^2^)	40 (SD 22)	42 (SD 10)	0.76
Indexed border zone area (cm^2^/m^2^)	21 (SD 10)	23 (SD 5)	0.66
Percent of border zone	14% (SD 4)	14% (SD 2)	0.83
Dense scar area (cm^2^)	43 (SD 26)	69 (SD 25)	0.03
Indexed dense scar area (cm^2^/m^2^)	22 (SD 13)	36 (SD 13)	0.03
Percent of dense scar	15% (SD 7)	23% (SD 9)	0.03

LVEF, left ventricular ejection fraction; RVEF, right ventricular ejection fraction; iRVEDV, indexed right ventricular end diastolic volume; iRVESV, indexed right ventricular end systolic volume; RV, right ventricular.

## Data Availability

Data will be available upon reasonable request (see Section 2).

## Supplementary Materials

The following supporting information can be downloaded at: https://www.mdpi.com/article/10.3390/jcm13133674/s1, Table S1: Comparison of the distribution of RV scar between ARVC cases and controls. Table S2: Diagnostic performance of scar location for the diagnosis of ARVC. Table S3: Scar parameters obtained with ADAS-3D from CT are compared between ARVC patients and controls, after having excluded the 4 patients with borderline diagnosis and their correspondent controls. Table S4: Scar parameters obtained with ADAS-3D from CT are compared between ARVC patients and controls, after having excluded the 4 patients with borderline diagnosis and their correspondent controls. Table S5: Correlation between ADAS parameters obtained from CT and other clinical/imaging variables in ARVC patients.
