# Supplementary material for: Diagnostic and Prognostic Value of Right Ventricular Fat Quantification from Computed Tomography in Arrhythmogenic Right Ventricular Cardiomyopathy
Source: J Clin Med. 2024 Jun 24;13(13):3674. doi: 10.3390/jcm13133674 (PMC11242505; doi:10.3390/jcm13133674)
Supplement: Supplementary file 1 [file jcm-13-03674-s001.zip › jcm-3059653-supplementary.pdf]

SUPPLEMENTARY MATERIAL

Supplementary Table S1. Comparison of the distribution of RV scar between ARVC cases and controls.

	**Controls**	**ARVC patients**	**p**
Number of segments with scar	1.6 (SD 1)	4.9 (SD 1.4)	<0.001
Scar in segment 1 (Basal anterior)	1 (4%)	16 (70%)	<0.001
Scar in segment 2 (Basal lateral)	1 (4%)	19 (83%)	<0.001
Scar in segment 3 (Basal inferior)	0 (0%)	11 (48%)	0.001
Scar in segment 4 (Mid anterior)	12 (52%)	21 (91%)	0.007
Scar in segment 5 (Mid lateral)	5 (21%)	16 (70%)	0.003
Scar in segment 6 (Mid inferior)	1 (4%)	5 (22%)	0.19
Scar in segment 7 (Apex)	17 (74%)	22 (96%)	0.1
Scar in segment 8 (Septum)	0 (0%)	2 (9%)	0.49
Scar in basal segments	2 (9%)	21 (91%)	<0.001
Scar in mid segments	12 (52%)	22 (96%)	0.002

Supplementary Table S2. Diagnostic performance of scar location for the diagnosis of ARVC.

	Sensitivity % (95% CI)	Specificity % (95% CI)	Efficiency %
>2 Segments with scar	96 (79-99)	87 (68-96)	91
Scar in segment 1 (Basal anterior)	70 (49-84)	96 (79-99)	83
Scar in segment 2 (Basal lateral)	83 (63-93)	96 (79-99)	89
Scar in segment 3 (Basal inferior)	48 (29-67)	100 (86-100)	74
Scar in segment 4 (Mid anterior)	91 (73-98)	48 (29-67)	70
Scar in segment 5 (Mid lateral)	70 (49-84)	78 (58-90)	74
Scar in segment 6 (Mid inferior)	22 (10-42)	96 (79-99)	59
Scar in segment 7 (Apex)	96 (79-99)	26 (13-47)	61
Scar in segment 8 (Septum)	9 (2-27)	100 (86-100)	54
Scar in basal segments	91 (73-98)	91 (73-98)	91
Scar in mid segments	96 (79-99)	48 (29-67)	72

Supplementary Table S3. Scar parameters obtained with ADAS-3D from CT are compared between ARVC patients and controls, after having excluded the 4 patients with borderline diagnosis and their correspondent controls.

	**ARVC patients**	**Controls**	**P**
Age	53 (SD 12)	54 (SD 9)	0.69
Sex (females)	7 (37%)	7 (37%)	1
RV area (cm2)	278 (257-344)	168 (152-198)	<0.001
Indexed RV area (cm2/m2)	157 (148-178)	95 (79-102)	<0.001
RV scar area (cm2)	108 (SD 35)	11 (SD 7)	<0.001
Indexed RV scar area (cm2/m2)	58 (SD 16)	6 (SD 3)	<0.001
Percentage of RV scar (%)	36% (SD 9)	6% (SD 4)	<0.001
RV BZ area (cm2)	44 (SD 14)	8 (SD 5)	<0.001
Indexed RV BZ area (cm2/m2)	23 (SD 6)	4 (SD 3)	<0.001
Percentage of RV BZ (%)	14% (SD 2)	4% (SD 2)	<0.001
RV dense scar area (cm2)	64 (SD 28)	3 (SD 2)	<0.001
Indexed RV dense scar area (cm2/m2)	34 (SD 14)	2 (SD 1)	<0.001
Percentage of RV dense scar (%)	22% (SD 9)	2% (SD 1)	<0.001
RV area Min-Max (cm2)	191-547	127-250	
Indexed RV area Min-Max (cm2/m2)	118-254	71-131	
RV scar area Min-Max (cm2)	50-174	1.4-24	
Indexed RV scar area Min-Max (cm2/m2)	32-88	0.7-13	
Percentage of RV scar Min-Max	22%-53%	0.6%-13%	
RV BZ area Min-Max (cm2)	24-79	1.2-18	
Indexed BZ area Min-Max (cm2/m2)	15-38	0.6-10	
Percentage of RV BZ Min-Max	11%-20%	0.5%-10%	
RV dense scar area Min-Max (cm2)	20-108	0.2-8	
Indexed RV dense scar Min-Max (cm2/m2)	13-61	0.1-4	
Percentage of RV dense scar Min-Max	8%-39%	0.09%-4%	
RV, right ventricular; BZ, border zone; Min-Max, minimum and maximum value.			

Supplementary Table S4. Scar parameters obtained with ADAS-3D from CT are compared between ARVC patients and controls, after having excluded the 4 patients with borderline diagnosis and their correspondent controls.

	**ARVC patients (borderline criteria)**	**Controls**	**P**
Age	56 (SD 17)	55 (SD 15)	0.95
Sex (females)	1 (25%)	1 (25%)	1
RV area (cm2)	271 (SD 41)	199 (SD 55)	0.08
Indexed RV area (cm2/m2)	136 (SD 13)	104 (SD 17)	0.03
RV scar area (cm2)	177 (SD 37)	9 (SD 4)	0.01
Indexed RV scar area (cm2/m2)	38 (SD 17)	5 (SD 2)	0.008
Percentage of RV scar (%)	27% (SD 10)	4% (SD 1)	0.003
RV BZ area (cm2)	33 (SD 14)	7 (SD 4)	0.01
Indexed RV BZ area (cm2/m2)	17 (SD 6)	4 (SD 2)	0.006
Percentage of RV BZ (%)	12% (SD 4)	3% (SD 2)	0.005
RV dense scar area (cm2)	44 (SD 26)	2 (SD 1)	0.02
Indexed RV dense scar area (cm2/m2)	21 (SD 13)	1 (SD 0.4)	0.02
Percentage of RV dense scar (%)	15% (8-23)	1% (0.7-1.4)	0.02
RV area Min-Max (cm2)	216-309	129-260	
Indexed RV area Min-Max (cm2/m2)	126-154	84-122	
RV scar area Min-Max (cm2)	37-122	4-14	
Indexed RV scar area Min-Max (cm2/m2)	23-61	2.6-7	
Percentage of RV scar Min-Max	17%-40%	3%-6%	
RV BZ area Min-Max (cm2)	20-45	3-12	
Indexed BZ area Min-Max (cm2/m2)	9-22	2-6	
Percentage of RV BZ Min-Max	7%-15%	2%-6%	
RV dense scar area Min-Max (cm2)	13-77	1-3	
Indexed RV dense scar Min-Max (cm2/m2)	8-39	0.6-1.5	
Percentage of RV dense scar Min-Max	6%-25%	0.8%-4%	
RV, right ventricular; BZ, border zone; Min-Max, minimum and maximum value.			

Supplementary Table S5. Correlation between ADAS parameters obtained from CT and other clinical/imaging variables in ARVC patients.

	**Correlation coefficient (95% CI)**	**P**
**Non-indexed RV parameters**		
RV scar area VS Age	0.01(-0.41 to 0.42)	0.97
RV BZ area VS Age	0.1 (-0.48 to 0.34)	0.71
RC dense scar area VS Age	0.05 (-0.37 to 0.45)	0.81
RV area VS RVEDV	0.95 (0.89 to 0.98)	<0.001
RV scar area VS RVEF	-0.56 (-0.8 to -0.17)	0.008
RV BZ area VS RVEF	-0.52 (-0.77 to -0.11)	0.02
RV dense scar area VS RVEF	-0.48 (-0.76 to -0.06)	0.03
RV scar area VS TAPSE	-0.42 (-0.71 to 0.004)	0.053
RV BZ area VS TAPSE	-0.28 (-0.62 to 0.17)	0.22
RV dense scar area VS TAPSE	-0.41 (-0.71 to 0.02)	0.06
RV scar area VS RVEDV	0.78 (0.54 to 0.9)	<0.001
RV BZ area VS RVEDV	0.92 (0.81 to 0.97)	<0.001
RV dense scar area VS RVEDV	0.57 (0.2 to 0.8)	0.005
RV scar area VS Number of negative T waves in precordial leads	0.65 (0.32 to 0.84)	<0.001
RV BZ area VS Number of negative T waves in precordial leads	0.43 (0.03 to 0.72)	0.04
RV dense scar area VS Number of negative T waves in precordial leads	0.63 (0.29 to 0.83)	0.001
**Indexed RV parameters**		
Indexed RV area indexed VS iRVEDV	0.92 (0.81 to 0.97)	<0.001
Indexed RV scar area VS RVEF	-0.58 (-0.81 to -0.21)	0.004
Indexed RV BZ area VS RVEF	-0.52 (-0.78 to -0.12)	0.02
Indexed RV dense scar area VS RVEF	-0.5 (-0.77 to -0.08)	0.02
Indexed RV scar area VS TAPSE	-0.46 (-0.74 to -0.05)	0.03
Indexed RV BZ area VS TAPSE	-0.32 (-0.65 to 0.12)	0.15
Indexed RV dense scar area VS TAPSE	-0.43 (-0.72 to -0.01)	0.046
Indexed RV scar area VS iRVEDV	0.72 (0.43 to 0.88)	<0.001
Indexed RV BZ area VS iRVEDV	0.91 (0.8 to 0.96)	<0.001
Indexed RV dense scar area VS iRVEDV	0.48 (0.08 to 0.75)	0.02
Indexed RV scar area VS Number of negative T waves in precordial leads	0.69 (0.39 to 0.86)	<0.001
Indexed RV BZ area VS Number of negative T waves in precordial leads	0.44 (0.04 to 0.73)	0.03
Indexed RV dense scar area VS Number of negative T waves in precordial leads	0.65 (0.33 to 0.84)	<0.001

**Scar Percentages**		
Percentage of RV scar VS RVEF	-0.5 (-0.76 to -0.08)	0.02
Percentage of RV BZ VS RVEF	-0.47 (-0.75 to -0.05)	0.03
Percentage of RV dense scar VS RVEF	-0.4 (-0.71 to 0.04)	0.07
Percentage of RV scar VS TAPSE	-0.34 (-0.67 to 0.1)	0.12
Percentage of RV BZ VS TAPSE	-0.13 (-0.52 to 0.31)	0.56
Percentage of RV dense scar VS TAPSE	-0.33 (-0.66 to 0.1)	0.13
Percentage of RV scar VS iRVEDV	0.4 (-0.02 to 0.7)	0.06
Percentage of RV BZ VS iRVEDV	0.73 (0.44 to 0.88)	<0.001
Percentage of RV dense scar VS iRVEDV	0.21 (-0.22 to 0.59)	0.33
Percentage of RV scar VS Number of negative T waves in precordial leads	0.49 (0.1 to 0.75)	0.02
Percentage of RV BZ VS Number of negative T waves in precordial leads	0.17 (-0.26 to 0.54)	0.43
Percentage of RV dense scar VS Number of negative T waves in precordial leads	0.49 (0.1 to 0.75)	0.02
